# Advances in the Assessment of Coronary Artery Disease Activity with PET/CT and CTA

**DOI:** 10.3390/tomography9010026

**Published:** 2023-02-01

**Authors:** Jacek Kwiecinski, Rafal Wolny, Alicja Chwala, Piotr Slomka

**Affiliations:** 1 Department of Interventional Cardiology and Angiology, National Institute of Cardiology, 04-628 Warsaw, Poland; 2 Department of Medicine (Artificial Intelligence in Medicine), Imaging, and Biomedical Sciences, Cedars-Sinai Medical Center, Los Angeles, CA 90048, USA

**Keywords:** coronary artery disease, ^18^F-sodium fluoride, coronary positron emission tomography, coronary computed tomography angiography, pericoronary adipose tissue attenuation

## Abstract

Non-invasive testing plays a pivotal role in the diagnosis, assessment of progression, response to therapy, and risk stratification of coronary artery disease. Although anatomical plaque imaging by computed tomography angiography (CTA) and ischemia detection with myocardial perfusion imaging studies are current standards of care, there is a growing body of evidence that imaging of the processes which drive atherosclerotic plaque progression and rupture has the potential to further enhance risk stratification. In particular, non-invasive imaging of coronary plaque inflammation and active calcification has shown promise in this regard. Positron emission tomography (PET) with newly-adopted radiotracers provides unique insights into atheroma activity acting as a powerful independent predictor of myocardial infarctions. Similarly, by providing a quantitative measure of coronary inflammation, the pericoronary adipose tissue density (PCAT) derived from standard coronary CTA enhances cardiac risk prediction and allows re-stratification over and above current state-of-the-art assessments. In this review, we shall discuss the recent advances in the non-invasive methods of assessment of disease activity by PET and CTA, highlighting how these methods could improve risk stratification and ultimately benefit patients with coronary artery disease.

## 1. Introduction

Coronary artery disease (CAD) remains the leading cause of morbidity and mortality worldwide [1]. As recommended by societal guidelines, in clinical practice, the work-up of patients with suspected or established coronary atherosclerosis is based on non-invasive imaging [2,3]. In particular, anatomical plaque imaging by computed tomography angiography (CTA) and ischemia detection with myocardial perfusion imaging are the current standards of care [2,3]. Beyond these imaging targets, there is a growing body of evidence that depicting the processes which drive atherosclerotic plaque progression and rupture has the potential to further enhance risk stratification.

### The Paradigm of Vulnerable Plaque

For many decades, assessment of atherosclerosis has been based around the imaging of obstructive luminal stenosis; however, this approach has important limitations. As the vast majority of lesions responsible for myocardial infarction are not obstructive (cause non-significant luminal stenosis), they will not be detected by noninvasive stress testing and shall likely be not considered clinically relevant on anatomical imaging [4,5]. Importantly, we are now aware that particular morphological features of coronary lesions act as markers of high-risk plaques. Pathological studies have shown that plaques prone to rupture have a large necrotic core, thin fibrous cap, macrophage infiltration, angiogenesis, plaque hemorrhage, and microcalcifications [6,7]. Although these features can be imaged with computed tomography, observational studies have indicated that the sensitivity (32.8%) and positive predictive value (6.4%) for distinguishing patients at an increased risk of myocardial infarction is limited [8,9]. Consequently, new non-invasive methods for enhancing risk stratification in patients with stable coronary artery disease are needed. The latest data suggests that rather than focusing on the morphology of atherosclerotic plaque, quantifying the activity of the inflammatory and calcification response is particularly promising in this regard.

## 2. Positron Emission Tomography (PET) Imaging

Although traditionally the role of nuclear imaging in CAD was primarily the evaluation of the presence and extent of myocardial ischemia with single-positron emission tomography (SPECT), recent advances in positron emission tomography (PET) have allowed imaging beyond myocardial perfusion. By imaging the processes which are directly involved in plaque progression and destabilization, PET has the potential to provide important clinical insights at the point of care [10].

Tracking inflammation with specific molecular targets allows for direct visualization of inflammation within the atherosclerotic plaque. ^18^F-Fluorodeoxyglucose (FDG) PET imaging of atherosclerosis provides a reliable and reproducible measure of vascular inflammation, as it indicates increased metabolic activity of macrophages and probably also reflects contributions from local hypoxia and efficiency of tracer delivery via the microcirculation [11]. However, molecular imaging with ^18^F-FDG is limited by problems related to tracer uptake in the myocardium; despite optimal patient preparation, suppression of myocardial activity is achieved in approximately two-thirds of patients [12,13].

The solution to those limitations may be to apply other radiotracers which have been established for cancer imaging (^68^Ga-DOTATATE, ^11^C-PK11195, and ^18^F-fluoromethylcholine) [14,15,16]. These radiotracers might be more specific for vascular inflammation and better suited to atherosclerotic plaque imaging. In particular, ^68^Ga-DOTATATE, which binds to the somatostatin subtype-2 receptor (SST2) on the surface of activated macrophages, has shown hope in cardiovascular imaging. This PET tracer provides an opportunity for the assessment of generalized atherosclerotic disease activity and detailed information about the local plaque functional phenotype, thereby distinguishing the culprit from non-culprit coronary lesions [16].

## 3. ^18^F-Sodium Fluoride Coronary PET Imaging

Atherosclerotic plaques are characterized not only by an inflammation pattern but also with active calcification processes. Indeed, although coronary macrocalcifications are widely considered to be a hallmark of stable lesions, according to pathological studies, microcalcifications are a marker of plaque vulnerability [17]. Such small foci of hydroxyapatite (or the bone mineral), which are beyond the resolution of non-invasive CT imaging, can be depicted with ^18^F-sodium fluoride (NaF) PET [18,19].

Although initially, ^18^F-NaF was developed for imaging the bony skeleton, on whole-body PET scans, increased uptake of this radiotracer was found to be often detectable within atherosclerotic plaque [20]. This observation was confirmed in dedicated cardiovascular clinical and preclinical studies [21,22,23,24]. Dweck et al. demonstrated that in coronary artery disease, there is a strong correlation between ^18^F-NaF uptake and established coronary calcium, yet at the same time, 41% of patients with a high disease burden (coronary calcium scores above 1000) have no significant ^18^F-NaF uptake [24]. This suggested that ^18^F-NaF uptake provides different information, relating to metabolically active calcific plaque and developing microcalcifications. Moreover, this information seemed to be of clinical significance in relation to symptomatic status, prior adverse events, and cardiovascular risk scores.

**Figure 1:**
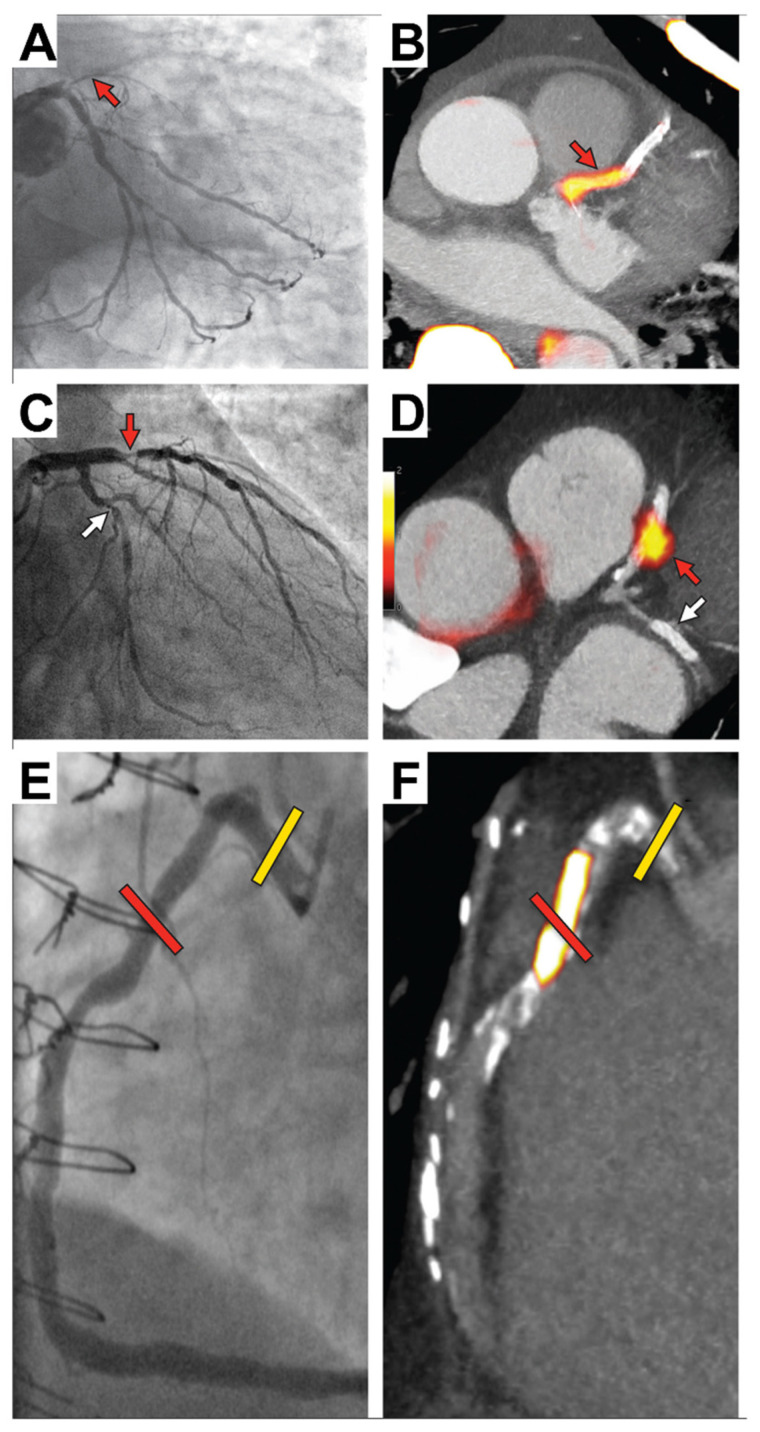
Focal ^18^F-NaF in patients with myocardial infarction and stable angina. Patient with acute myocardial infarction with (**A**) proximal occlusion (red arrow) of the left anterior descending artery on invasive coronary angiography and (**B**) intense focal ^18^F-NaF uptake (yellow-red) at the site of the culprit plaque (red arrow) on PET. Patient with anterior myocardial infarction with (**C**) culprit (red arrow; left anterior descending artery) and bystander non-culprit (white arrow; circumflex artery) lesions on invasive coronary angiography that were both stented during the index admission. Only the culprit lesion had increased ^18^F-NaF uptake on PET (**D**). In a patient with stable angina with previous coronary artery bypass grafting, invasive coronary angiography (**E**) showed non-obstructive disease in the right coronary artery. Corresponding PET scan (**F**) showed a region of increased ^18^F-NaF in the mid-right coronary artery and a region without increased uptake in the proximal vessel. This figure was originally published in the Lancet under the Creative Commons Attribution 4.0 International License (https://creativecommons.org/licenses/by/4.0/) (accessed on 31 December 2022) (Data from [31]).

Following these initial studies over the past 8 years, ^18^F-NaF has been applied to multiple cardiovascular conditions, including aortic stenosis, prosthetic valve degeneration, abdominal aortic aneurysm, peripheral vascular disease, and erectile dysfunction, and ^18^F-NaF consistently predicts disease progression and clinical events [25,26,27,28,29,30]. In the coronary arteries, ^18^F-NaF uptake can be localized to individual coronary lesions, identifying plaques with multiple adverse features as well as predicting unfavorable outcomes [18]. In an observational cohort study, Joshi et al. demonstrated that patients with a recent myocardial infarction have increased tracer activity in culprit lesions, suggesting that ^18^F-NaF PET could act as a predictor of adverse events (Figure 1) [31]. These encouraging observations lead to further studies which explored ^18^F-NaF in the populations of particular patients; provided data for enhancing acquisition, reconstruction, and postprocessing; and ultimately served for outcome analyses.

### 3.1. ^18^F-NaF Uptake and Adverse Plaque Morphology

In stable coronary artery disease, both invasive and non-invasive imaging studies have consistently shown that ^18^F-NaF uptake is frequently observed in high-risk lesions. Plaques with positive remodeling, a necrotic core, and microcalcifications on intravascular ultrasonography have been shown to present with high tracer activity [31,32]. On CT imaging, initial reports linked ^18^F-NaF uptake to partially calcified lesions. More recently, comprehensive CT studies have shown that approximately two thirds of stable patients with high-risk plaques have abnormal ^18^F-NaF uptake [33]. Although over 90% of low attenuation plaques (consistent with a necrotic core) are closely associated with increased tracer activity, only 55% of lesions with positive remodeling and 57% of those with spotty calcifications present with high ^18^F-NaF uptake [33]. Additionally, high tracer activity was also observed within lesions without adverse morphology. It was therefore concluded that there is a substantial discordance in morphological plaque characteristics and the activity of the disease (measured with ^18^F-NaF PET), suggesting that they provide complementary information.

### 3.2. Technical ^18^F-NaF Refinements

Due to the fact that microcalcifications comprise a small proportion of atherosclerotic plaques which, on their own, are small imaging targets compared to oncological lesions, coronary ^18^F-NaF PET is a challenging imaging modality [34]. The quality of the data can be further compromised by constant motion which occurs as a combined product of heart contractility and breathing. Luckily, beyond gating, which is the traditional method for offsetting such motion, we can now benefit from dedicated motion correction techniques [35,36,37,38,39]. It was demonstrated that by applying dedicated tools, the reproducibility of the measurements can be significantly improved. In a series of studies, Lassen et al. showed that rather than relying on complex equipment which tracks patient motion, accurate motion correction can be performed with data-driven approaches. This method enables offsetting for patient’s repositioning during the typically lengthy PET acquisition by evaluating the center of mass of the acquired PET list-mode file data, thus measuring the motion vector fields and co-registration of data with a rigid three-parameter translation based on the center of mass assessment [37]. Further gains in image quality can be achieved by performing image acquisitions at a longer (3 h) delay from tracer injection [40]. Recently, to streamline image analysis, artificial intelligence approaches have been harnessed to facilitate co-registration of CT and PET images [41]. Given the aforementioned advances, along with dedicated software for analysis and imaging protocols which have been validated extensively, ^18^F-NaF coronary PET is now becoming a technology which can be easily implemented across PET labs [42,43,44,45,46].

### 3.3. ^18^F-NaF Uptake as a Predictor of Myocardial Infarction

**Figure 2:**
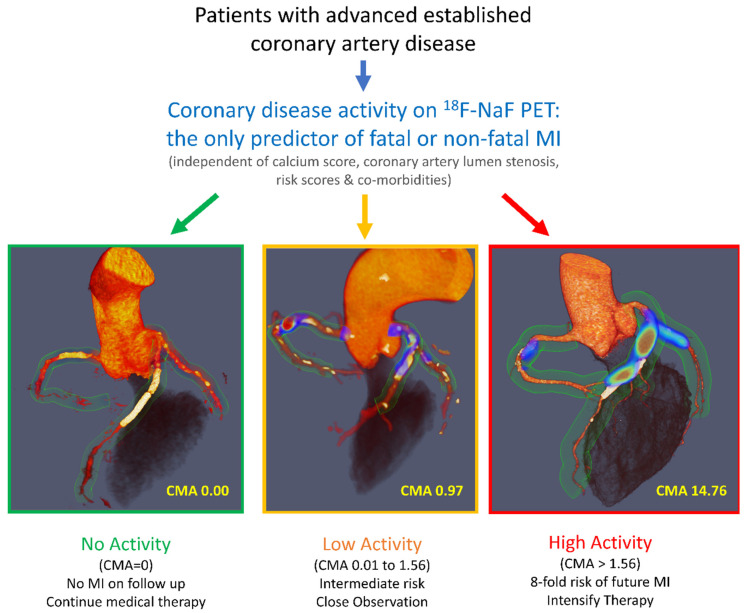
^18^F-sodium fluoride positron emission tomography as a marker of disease activity in the coronary arteries is a predictor of fatal or non-fatal myocardial infarction (MI) in patients with established coronary artery disease.^18^F-NaF PET can be used to measure disease activity across the coronary vasculature and to stratify patients into those with no, low, and high disease activity. Figure 2 is reprinted from [47], with permission from Elsevier.

The data collected in the context of multiple observational studies have been recently leveraged for post-hoc analyses which demonstrated that coronary ^18^F-NaF PET is a powerful prognostic tool for predicting myocardial infarction in patients [47]. In 293 patients with advanced established coronary artery disease, measuring disease activity with PET outperformed all other established predictors, including the presence of comorbidities; risk scores; coronary calcium scoring; and the presence, severity, and extent of coronary artery disease (Figure 2). These findings have been confirmed in further studies [48,49]. ^18^F-NaF might, therefore, provide an important clinical tool in a patient population in which risk stratification is currently suboptimal. Importantly, high ^18^F-NaF coronary uptake is associated with a several-fold increase in the risk of myocardial infarction in subjects on secondary preventative therapies with almost universal prescription of aspirin and statins. These patients might, therefore, be suitable for advanced medical therapies, including PCSK9 or interleukin 1-beta inhibition, with ^18^F-NaF PET providing the risk stratification tool that many have advocated for as a means of targeting these expensive drugs to those patients at the greatest risk.

Given that in parallel to PET, coronary CT angiography has also evolved rapidly over the past years, it could be argued that there is no need for disease activity assessments with ^18^F-NaF. This hypothesis was recently tested in a multimodality study which showed that for optimal risk stratification, it is necessary to consider the wealth of information provided by both PET and CT (anatomical) imaging [50]. It was concluded that although the data provided by these two imaging modalities are correlated, machine-learning modelling can be employed for combining this information [50].

Beyond prediction of myocardial infarction ^18^F-NaF PET can also predict progression of the disease [51,52,53]. In three independent studies, increased ^18^F-NaF uptake was associated with more rapid progression of coronary atherosclerotic calcification. This finding was consistent across a range of measures of calcification and whether this was considered on a per-patient or per-segment basis. This holds true not only for native coronary arteries prior to revascularization, but also for patients following coronary artery bypass grafting [54]. Although microcalcification activity is not a dominant feature of graft vasculopathy, native coronary arteries that have been bypassed demonstrate increased disease activity and more rapid disease progression than non-bypassed arteries, an observation that appears to be independent of the baseline atherosclerotic plaque burden [54].

## 4. Coronary Computed Tomography Angiography

Despite recent technical refinements and the accumulation of scientific evidence, the access to cardiac PET remains limited. This raises an important question as to whether similar data on CAD activity can be obtained using other, potentially more accessible imaging modalities. Coronary computed tomography angiography (CTA) is one such promising method. Along with remarkable technical advances in multidetector scanner designs, CTA represents a significant step forward from conventional invasive angiography through its ability to visualize not only the lumen, but more importantly, the coronary vessel wall and atherosclerotic plaques [55]. Its high diagnostic accuracy in detecting significant coronary stenoses has paved the way to its implementation as a first-line diagnostic tool in a growing number of patients evaluated for the presence of CAD according to societal guidelines [2]. Due to its excellent negative predictive value for CAD detection, CTA frequently serves as a “gatekeeper” for invasive coronary angiography, leading to a decrease in the number of unnecessary catheterizations and reducing the time to diagnosis and discharge [56,57]. Importantly, its application has recently been extended to several other fields, including but not limited to plaque phenotyping and mapping of coronary inflammation.

### 4.1. CTA for Detection of Vulnerable Plaque

Intravascular ultrasound and optical coherence tomography constitute the gold standard for in vivo imaging of coronary plaques in humans, and these techniques are able to detect features of vulnerability, such as a thin fibrous cap, positive remodeling, low attenuation/lipid component, and small luminal area, which all are predictive of future myocardial infarctions [8]. However, their invasive nature carries a low, yet non-negligible risk of complications. Thus, especially in patients with non-obstructive CAD for the purpose of risk stratification, there was an unmet need for non-invasive and cost-sparing alternatives. Seminal studies by Hoffmann et al. and Motoyama et al. have shown that coronary plaques in patients presenting with acute coronary syndromes compared with plaques in stable patients possess distinct features on CTA [58,59]. Those features include a low-attenuation component (<30 HU being the most frequently applied cutoff), positive remodeling, and spotty calcifications, as well as the subsequently described “napkin-ring” sign [60]. The presence of such “high risk” features in patients with stable coronary artery disease was shown to predict future myocardial infarctions [9]. Semiautomated volumetric quantification of the non-calcified, low-attenuation coronary plaque component was another refinement which allowed for improved prediction of myocardial infarction in patients with stable chest pain who were enrolled in the large SCOT-HEART trial [61,62]. Despite those advances, still a significant number of acute coronary events can be ascribed to lesions without CT-detected high-risk features [63]. Moreover, the risk reclassification potential arising from high-risk plaque detection is greatest in lower risk groups, such as younger patients, women, and those with non-obstructive CAD [64]. This fact constitutes the rationale for further research on the improvement of risk stratification in this population.

### 4.2. CTA for Assessment of Coronary Inflammation

**Figure 3:**
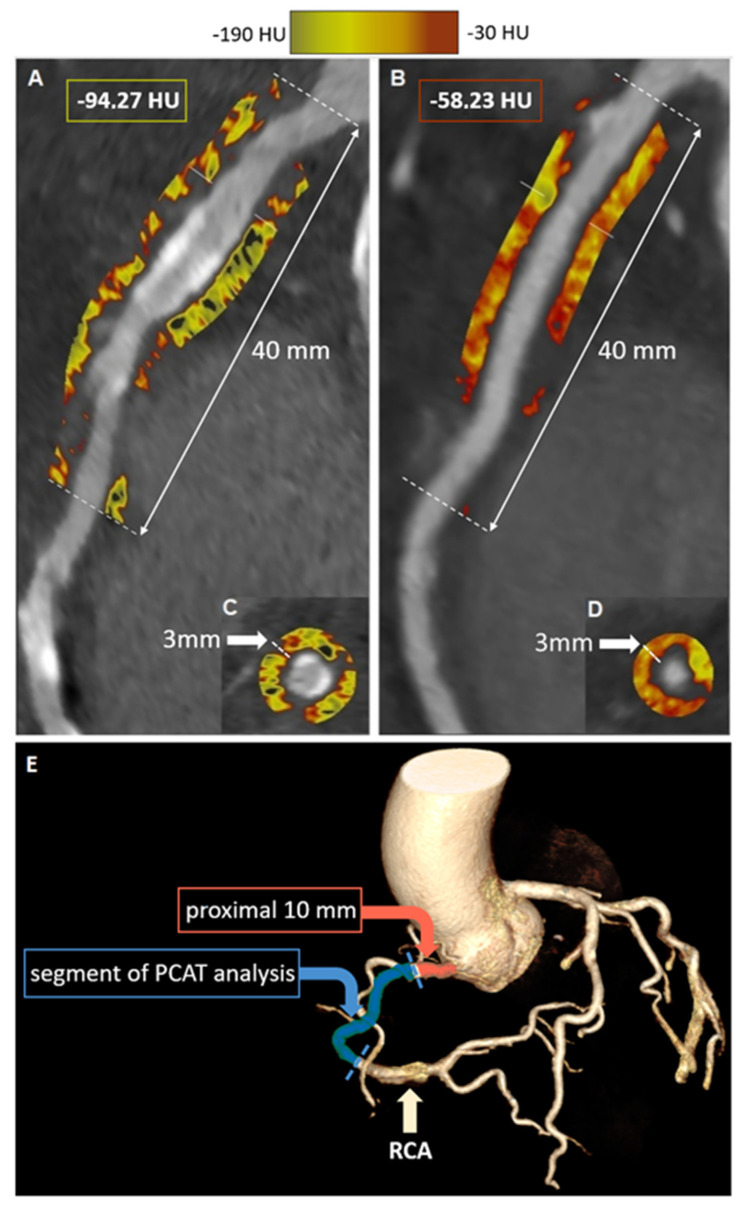
Examples of semi-automated analysis of CTA-derived PCAT attenuation. Two examples of semi-automated PCAT analysis of the right coronary artery using Autoplaque® software (Cedars Sinai Medical Center, Los Angeles, CA, USA). The first case ((**A**)—longitudinal view, (**C**)—short-axis view) shows a low PCAT of −94.27 HU, and the second case ((**B**)—longitudinal view, (**D**)—short-axis view) shows a high PCAT of −58.23 HU, suggesting increased perivascular inflammatory activity. (**E**)—schematic representation of the principle of PCAT analysis around the RCA involving a 40-milimeter section of the artery excluding the proximal 10 millimeters.

Recently, CTA was repurposed for non-invasive assessment not only of the anatomy of the coronary tree but also of coronary inflammation. The concept was first introduced by Antonopoulos et al., who took advantage of the fact that CT attenuation of perivascular tissue depends on the balance of its lipid and water content [65]. They have shown that inflamed human vessels release cytokines that inhibit maturation of preadipocytes to adipocytes, thus preventing lipid accumulation in the perivascular space. This shift in adipocyte tissue lipid content leads to a detectable change in CT attenuation, which can be used as an imaging biomarker for pericoronary inflammation. They developed a novel imaging metric (FAI = Fat Attenuation Index) and validated it against 18-F FDG PET imaging. In brief, FAI quantifies a CT attenuation in concentric 1 mm layers of perivascular tissue around the human arterial wall, capturing the gradient of perivascular weighted CT attenuation with increasing distance from the arterial wall [66]. The pericoronary adipose tissue density (PCAT) is a similar metric of perivascular inflammation and is defined as the average CT attenuation, expressed in Hounsfield units, of adipose tissue within the defined volume of interest [67]. Regardless of the applied methodology, quantification of the pericoronary adipose tissue attenuation has been semi-automated and can be easily derived retrospectively from any standard CTA dataset using dedicated software (Figure 3).

**Figure 4:**
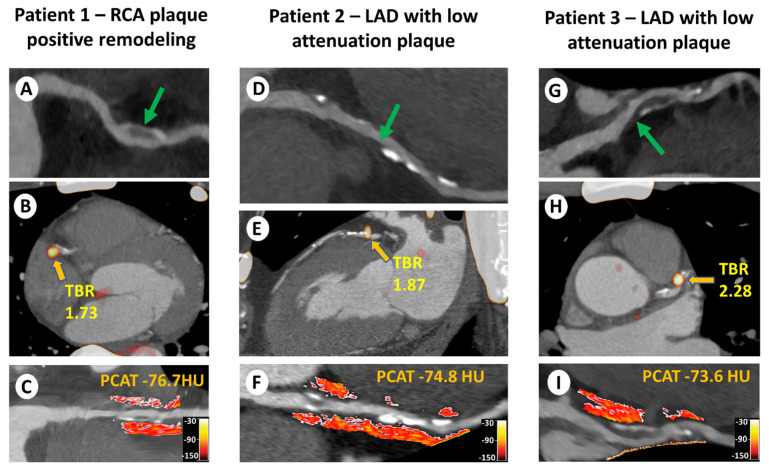
Case illustrations of coronary CTA and assessment of 18F-NaF uptake and PCAT attenuation in high-risk plaques. Patient 1: 53-year-old man with a (**A**) right coronary arterial (RCA) plaque with positive remodeling (green arrow); (**B**) focal 18F-sodium fluoride (18F-NaF) uptake with increased target-to-background ratio (TBR) of 1.73; and (**C**) increased peri-coronary adipose tissue (PCAT) attenuation (mean PCAT density: −76.7 Hounsfield units [HU]). (**D**) Patient 2: 66-year-old man with a (**E**) left anterior descending (LAD) lesion with low attenuation plaque (yellow arrow); (**F**) focal 18F-NaF uptake with increased TBR of 1.87; and increased PCAT attenuation (mean PCAT density: −74.8 HU). (**G**) Patient 3: 54-year-old human with an LAD lesion with low attenuation plaque (green arrow); (**H**) focal 18F-NaF uptake with increased TBR of 2.28; and (**I**) increased PCAT attenuation (mean PCAT density: −73.6 HU). CTA = computed tomography angiography. Figure reprinted from [68], with permission from Elsevier.

The aforementioned methods have already been widely validated. In a seminal study, Antonopoulos et al. measured FAI around the proximal right coronary artery in 273 patients with CAD and found significantly higher values in patients with CAD compared with healthy individuals. FAI was also higher around culprit lesions in patients with acute coronary syndromes compared with non-culprit plaques, and it progressively decreased over time after the acute event. These findings were further expanded by Goeller et al., who showed that pericoronary CT attenuation was increased around culprit lesions compared with non-culprit lesions of patients with ACS and lesions in matched controls [67]. Pericoronary adipose tissue attenuation has also been correlated with other imaging parameters associated with plaque vulnerability and vascular dysfunction (Figure 4). In a multimodality imaging study of individuals with stable CAD and high-risk coronary plaques who underwent both CTA and ^18^F-NaF PET, PCAT was significantly higher in patients with high ^18^F-NaF uptake compared to the low-uptake group [68].

Given its high inter- and intraobserver reproducibility, as well as its hardware-independence, PCAT holds promise as a potential tool for monitoring longitudinal changes in perivascular inflammation in response to medication [69]. In a study of patients with moderate to severe psoriasis who underwent serial CTA imaging, biologic therapy was associated with a significant decrease in pericoronary inflammation at one year compared with patients not receiving such treatment [70]. Along with the advent of inflammation-targeted drugs such as colchicine (COLCOT, LoDoCo2) and canakinumab (CANTOS), which showed significant clinical efficacy in randomized trials of patients with CAD, serial measurement of perivascular inflammation may serve as a surrogate endpoint for further research in this field [71,72,73].

### 4.3. CTA-Detected Perivascular Inflammation and Clinical Outcomes

Ultimately, PCAT was shown to be associated with clinical prognosis in patients with CAD. In the CRISP-CT study among 3912 patients undergoing CTA for clinical indications, higher FAI measured around the proximal right coronary artery was an independent predictor of cardiac mortality [74]. The optimal FAI cut-off value indicating increased risk was −70.1 HU. These results were confirmed by van Diemen et al. on a cohort of 539 patients with suspected CAD, in whom PCAT measured around the proximal right coronary artery above scanner-specific thresholds was not only an independent predictor of death and myocardial infarction at five-year follow-up but also provided incremental prognostic value beyond clinical characteristics, calcium score, obstructive CAD, high risk plaque features, quantitative plaque volume, and myocardial ischemia [75]. Most recently, in a post-hoc analysis of 1697 patients enrolled in the prospective SCOT-HEART trial, higher PCAT around the proximal right coronary artery was predictive of myocardial infarction at a median follow-up of 4.7 years with an optimal cut-off value of −70.5 HU [76]. Adding PCAT to the low attenuation plaque burden significantly improved risk prediction in this population. This observation is in line with the study which demonstrated that assessments of plaque activity with PET can be further enhanced with in-depth anatomical coronary plaque volume and composition measurements. It can therefore be concluded that although disease activity assessments clearly improve risk stratification, it is necessary to consider them along with anatomical imaging.

## 5. Future Directions

The assessments of disease activity can be employed beyond the imaging of coronary atherosclerosis. Although there are a handful of studies which evaluated the utility of measuring the adipose tissue density (analogous to PCAT) outside of the coronary vasculature, ^18^F-NaF uptake on PET has been shown to act as a powerful predictor of disease progression and adverse outcomes across a wide range of cardiovascular conditions. These include valvular heart disease, atherosclerosis across different vascular beds (the aorta, lower limb, cervical, and penile arteries) and abdominal aorta aneurysmal disease. In aortic stenosis—the most common valvular condition—^18^F-NaF predicts the progression of disease and can also be leveraged for risk stratification in patients who underwent surgical or transcatheter valve replacement with a bioprosthesis. Each year, over 400,000 patients with severe symptomatic disease undergo valve replacement, receiving a tissue valve (bioprosthesis) which is prone to degeneration. For decades, we have lacked robust tools for the prediction of bioprosthesis failure, and the disease was typically detected at an advanced stage when re-intervention is a high-risk undertaking. ^18^F-NaF PET addresses this important gap by enabling the differentiation of those subjects who are at an increased risk of bioprosthesis failure. In a series of observational studies, we have demonstrated that increased ^18^F-NaF uptake in the bioprosthetic valve leaflets provides an early indication of valve degeneration and a more powerful prediction of its subsequent dysfunction than valve age, cardiovascular comorbidities, and imaging data provided by echocardiography and CT [26,27].

In the future, such efforts to elucidate the potential of non-invasive disease activity assessments and to validate their utility across various cardiovascular conditions should be continued. For further research on coronary artery disease, it is equally important to establish optimal allocation of disease activity assessments. Emerging research suggests that it could serve for monitoring the response to therapy [77,78]. Consequently, it appears that disease activity imaging could be employed for targeting emerging expensive therapies (such as potent lipid lowering or anti-inflammatory medication) in patients at the greatest risk of adverse events.

## 6. Conclusions

In summary, the presented state-of-the-art overview of the non-invasive assessment of coronary artery disease activity with CTA and PET imaging illustrates the unprecedented recent development of both methods. The available evidence on the accuracy of diagnosis of vulnerable plaques, good correlation with intravascular and pathological studies, high reproducibility, and clinical validation for prediction of hard endpoints in well-defined cohorts of patients with long-term clinical outcomes suggest that both CTA-derived pericoronary adipose tissue density and ^18^F-NaF PET are ready for wider clinical application.

## Data Availability

No new data were created or analyzed in this study. Data sharing is not applicable to this article.
